# Longitudinal pharmacoepidemiological and health services research for substance users in treatment: protocol of the Belgian TDI-IMA linkage

**DOI:** 10.1186/s13690-017-0249-x

**Published:** 2018-01-08

**Authors:** Luk Van Baelen, Karin De Ridder, Jérôme Antoine, Lies Gremeaux

**Affiliations:** Department of Public Health and Surveillance, Scientific Institute of Public Health, Brussels, Belgium, Rue Juliette Wytsmanstraat, 14, 1050 Brussels, Belgium

**Keywords:** Substance use disorders, Pharmacoepidemiological data, Health services, Belgium, Protocol

## Abstract

**Background:**

Not much is known about the health seeking behavior of people with substance use disorders before they enter specialized treatment and afterwards. This paper explains in detail the protocol that has been followed to establish the Belgian TDI-IMA-database, which is linking two separate databases: the Treatment Demand Indicator (TDI) and the database of the Intermutualistic Agency (IMA). The Treatment Demand Indicator is measuring incidence of people with substance use disorders entering drug treatment. The IMA-database covers data, collected in the framework of the compulsory Belgian health care and benefits insurance program, on reimbursed medication and the use of reimbursed health services. The linkage results in pharmacoepidemiological and health service data for people who were in treatment for substance use disorders and for a group of comparators.

**Methods:**

The TDI-database was linked to the IMA-database for the period between 01/01/2008 and 31/12/2017, based on the national identification number of patients who have been in alcohol or drug treatment between 01/01/2011 and 31/12/2014. Through this linkage, pharmacoepidemiological and health service data became available for at least 3 years before the first registered episode in the TDI-database till at least 3 years after the first episode. For each person in TDI four comparators, who were not in specialized treatment, were matched on age, sex and place of residence.

**Discussion:**

The TDI-IMA-database allows for an analysis of health seeking behavior and health care pathways of people before and after they entered specialized alcohol and drug treatment. The presented protocol could be used in other European countries to establish a linkage between existing health databases. This will allow for a better understanding of the health care needs of patients with substance use disorders.

## Background

The first countrywide electronic pharmacoepidemiological databases in Europe were developed in the beginning of the 1990’s in Scandinavia. These registers were created to collect information on dispensed medication and to conduct observational studies to better understand drug effects, as well as safety and cost-effectiveness of medication [1]. An example of such a database is the Danish Registry of Medicinal Product Statistics [2] which contains information for the entire Danish population since 1994 about age, sex and municipality of the drug user, quantity of and expenditures for prescribed drugs, the practice code of the prescriber and the practice code of the dispensing pharmacy. Each person is identified by a unique encrypted personal number, which enables the linkage to other registers such as databases with hospitalization records, mortality registers or socio-demographic registers, e.g. Torstensson et al. [3]. Although the last decades have seen an increase in the number of health related and socio-demographic databases, such cross-linkage of data remains rare.

At the same time other EU countries developed databases following an integrated approach, characterized by the collection of information on patients’ socio-demographics and prescribed drugs as well as all other types of health care events. Examples are the UK General Practice Research Database [4], derived from medical records and in place since 1994, and the German Statutory Health Insurances database [5]. The scope of these registers is not limited to prescribed medication but includes also the incidence and prevalence of common illnesses [6]. The setup allows for the analyses of patterns of treatment and outcome.

Databases for pharmacoepidemiological and health services research have been used to analyze health events in the general population but also in subpopulations with specific diseases such as diabetes [6], mental health problems [7], cardiovascular diseases [3] or asthma [8]. In the research field on alcohol and illicit drug use, pharmacoepidemiological and health services databases have been analyzed to better answer the needs of people with problematic drug use, to track the pathway to recovery, to examine the functioning of drug treatment centers, to provide empirical evidence for the effectiveness of drug treatment and to show the impact of policy changes [9, 10]. In Belgium, such research has been lacking so far. To fill this gap, two separate databases were linked, the Treatment Demand Indicator (TDI) and the databases of the Intermutualistic Agency (IMA).

TDI is an epidemiological indicator collected in a standardized way in all 28 member states of the European Union, as well as Turkey and Norway on behalf of the European Monitoring Centre for Drugs and Drug Addiction (EMCDDA) [11]. In Belgium registration has started on January 1st, 2011, based on the protocol agreement of December 12th, 2005 [12]. TDI registers the initiation of specialized drug treatment episodes and allows for a structured exchange of information with national and international drug agencies [11]. Data on patients are self-reported and collected in places such as psychiatric hospitals, centers for mental health or therapeutic communities. They reflect a situation at one point in time, often the situation on the day of admission. As cross-sectional data, the register is measuring the incidence of people entering drug treatment because of substance use disorders. However, for most of these patients, this demand is part of a process that may have been going on for many years. TDI on its own gives only limited insight in health seeking behavior of people before they enter specialized drug treatment and it lacks information on post-treatment follow-up.

The Intermutualistic Agency (IMA) was founded by law in 2002 to integrate into one database all information as collected by the seven existing health insurance agencies in Belgium. This database covers data on reimbursed medication and reimbursement for the use of health services, collected in the framework of the compulsory Belgian health care and benefits insurance program. In accordance with the law of 14 July 1994, membership to a health insurance agency is obligatory in Belgium when someone starts working or gets an allowance, or when he or she reaches the age of 25 and is still studying. The IMA database consists of three main sources: (1) official administrative population data, (2) information on reimbursed health services provided by hospitals, general practitioners, specialists, nurses or other medical care providers and (3) information on reimbursed medication sold in public pharmacies. However in turn, it does not allow for identification of people with substance use disorders.

The linkage of both TDI and IMA databases facilitates a better understanding of the care pathways of patients before, during and after specialized drug treatment. This means that it becomes possible: (1) to analyze and compare the use of health services by people with substance use disorders; (2) to analyze and compare the prescription of medication for people with substance use disorders; (3) to compare information on patients with a group of peers who were not in specialized alcohol and drug treatment; (4) to estimate the coverage of the TDI-database and to validate the database by comparing data in TDI on for instance socio-demographic variables or substitution treatment with corresponding data from the IMA-database.

## Methods/design

### Case definition

Cases were selected based on patients’ first registration in the TDI-database between 01/01/2011 and 31/12/2014, following the case definition used by Antoine and colleagues for the Belgian TDI registration (protocol 2.0) [11]: in TDI information is collected (a) on every treatment episode (b) started by a person (c) in a treatment center (d) for his or her alcohol or illicit drug use.

(a) An episode is defined as the period between the start of the treatment, which is the first face-to-face contact between a professional and the patient, and the end of activities in the context of the program prescribed. In outpatient settings this end of the episode occurs when the patient stops attending treatment for a period longer than 6 months, whereas in inpatient settings, it is defined as the moment when the patient leaves the center and no further admission is foreseen. However, data only show an image at the start of the episode and no information is available about the duration of the episode. Patients also have the right to refuse registration. If in the period 2011 to 2014 patients were admitted to treatment more than once, data from the start of the first episode were used for the current linkage. Since some patients were in treatment before 2011, this means that this first episode does not necessarily correspond to the first treatment ever, nor does it mean that there have not been any other treatment episodes afterwards. Treatment is defined as any activity targeting a person with substance use problems directly in order to obtain results in terms of reducing or eliminating these problems.

(b) The registration concerns all individuals without any restriction, with the only condition that the patient should have had a face-to-face contact with a care giver for his or her substance use problem.

(c) Activities have to take place in a treatment center, which is defined as a facility or practitioner providing treatment for drug or alcohol addiction. It can be an outpatient or inpatient service, either specialized in addiction treatment or included in larger scale facilities targeting different groups of people, and sometimes but not always recognized within a convention of authorities such as the National Institute for Health and Disability Insurance (NIHDI) [11].

(d) Drug types that are registered are opioids, cocaine/crack, stimulants other than cocaine, hypnotics and sedatives, hallucinogens, volatile inhalants, cannabis and alcohol, and their subcategories.

### Linkage procedure TDI-IMA

The TDI-database was linked to the IMA-database for the period between 01/01/2008 and 31/12/2017, based on the national identification number (NIN) of the patient. This number is unique for every Belgian citizen and for other people living in Belgium with social security rights. It is used in TDI to avoid double counting over several treatment episodes and over different treatment centers. In TDI, the NIN was encrypted for the linkage by a trusted third party, eHealth. In IMA, the NIN was encrypted twice by a trusted third party, the Crossroads Bank for Social Security (CBSS). The linkage procedure has been established in line with all relevant national privacy rules [13].

Through this linkage, pharmacoepidemiological and health service data became available for at least 3 years before the first registered episode in the TDI-database till at least 3 years after the first episode. This time span not only allows analysis of short- and long-term effects of a specific treatment approach, but also the conducting of longitudinal analyses on pathways in health seeking behavior before and after specialized alcohol and drug treatment. The chosen reference period started in 2008 as the structure of the Belgian social security system changed fundamentally at that time. For instance, since 2008 all beneficiaries of the official health insurance agencies (including people who are working as independent contractor) have access to the same package of health care, including insurance for small risks.

An added asset of the linkage is the inclusion of a group of people who were not attending specialized alcohol and drug treatment between 2008 and 2014. This group was selected from the IMA-database and allows for comparative analyses. For every patient included in the TDI-database four comparators were drawn at random from the clients of the seven Belgian health insurance companies [14]. Cases and comparators were matched on sex, age and place of residence [15]. Sex and gender were used as basic matching variables. The matching on municipality was related to both the underlying regional differences in health care regulation and health care availability (with for instance geographically different access to specialized medical health care for substance use disorders) as well as socio-economical differences between patients in the different regions of Belgium. As a result, this matching procedure allows adjusting for confounders and at the same time it created a “comparative” group that is similar to the patients in TDI who are mainly men (71.8%) with an average age of 39.8 years [16].

Five exclusion criteria were applied in the matching procedure: (1) comparators passed away in the year in which the case had a first episode in treatment, (2) comparators used medication for opiate dependence, defined by the corresponding Anatomical Therapeutic Chemical Classification System Code (ATC-code: N07BC), between 2008 and 2014, (3) comparators used medication for alcohol dependence (ATC-code: N07BB) between 2008 and 2014, (4) comparators used opioid substitution medication (based on national nomenclature codes referring to extemporaneous preparations based on methadone) between 2008 and 2014 and (5) comparators attended a health service for alcohol or drug treatment (based on national nomenclature codes referring to treatment of substance use disorders) between 2008 and 2014. If it was not possible to select four comparators, the matching criteria were expanded to a 5 year age group instead of the same age (year) and to the arrondissement (an administrative level, grouping municipalities) instead of the place of residence (municipality) of the case.

### Registration and collection of variables

Participation of treatment centers to the TDI registration is constantly evolving over time due to obligations by the different Belgian authorities [12, 17]. During the period 2011–2014 mainly specialized treatment centers with a convention with the NIHDI, centers with an agreement from the Walloon region, centers of mental health in Flanders and a group of voluntary hospitals participated in the data collection. The participating TDI data providers can choose between two registration systems: an online registration module that allows for a case-by-case registration and a repository module that functions as a secured mailbox through which structured files can be submitted, containing a complete dataset for a given registration year [11]. At the start of every treatment episode a form has to be completed by a trained care giver of the participating treatment center during a face-to-face interview with the patient. Data are registered (1) about the treatment center (identification on the level of the center itself, on the level of a unit or program or satellite within the center, the type of program and its geographical location), (2) about the patient (socio-demographic data (sex, age and nationality) and socio-economic information (type of living accommodation, type of household, educational level, work and income situation)), and (3) about the treatment episode (the start date, history of previous treatment for substance use disorders, the main source of referral, his/her substitution treatment situation, substances used, patterns of use for the main primary substance and injecting status) [11]. After quality checks, performed in the registration module or after reception of the file from the repository module, data is stored in a database where each record corresponds to a treatment episode.

As said, the IMA database includes (1) administrative socio-demographic data, (2) information on reimbursed health services and (3) information on reimbursed medication sold in public pharmacies. For the IMA socio-demographic database the seven Belgian health insurance agencies collect data twice a year with specific demographic and socio-economic indicators [14]. This data is transferred to IMA, where one aggregated file of the seven health insurance agencies is created. It results in one file with individual socio-demographic indicators for 99% of the people living in Belgium during the reference year. In this database information is registered about age, sex, vital status (alive or passed away in the year of registration), patient’s status concerning specific social categories (widowhood, invalidity category, chronic illness, incapacity benefits, allowance for people with invalidity…), information about rights concerning maximum costs for medical care of the patient, work status and province, arrondissement and degree of urbanization of the place where the patient is living [14].

For the IMA health services database, indicators are collected for all health care related expenses which are reimbursed according to the compulsory insurance [18]. Data are collected either at the counter of the health insurance agency, either through third parties such as hospitals, groups of nurses, homes for elderly, or together with pharmaceutical data. It consists of information about the unique code of the prescriber, the unique code of the service provider, professional qualification of the prescriber/service provider, medical service by nomenclature code, subcategories of medical services, day, month and year of service delivery, type and qualification of the health institution, amount of costs that are reimbursed by the health insurance agency, amount of costs paid by the patient and supplements linked to medical services, and ATC-code for medication that is provided through hospital pharmacies [18].

The IMA pharmaceutical database consists of all the expenditures for medication which are reimbursed according to the compulsory insurance and which are not distributed through hospital pharmacies [19]. The collection is done through the accounts department of the pharmacies and delivered electronically. Information is registered about the unique code of the prescriber, the unique code of the service provider, qualification of the prescriber/service provider, day, month and year of delivery of the product, a unique national code number for every product (CNK-code), ATC-code, Defined Daily Doses (DDDs), Quantities per package (QPP), Quantities per Unit (QPU), medical service by nomenclature code, amount of costs that are reimbursed by the health insurance agency, amount of costs paid by the patient and supplements linked to medical services [19].

### Analysis and reporting

Descriptive statistical analysis was performed using SAS software version 9.3 (SAS Institute Inc., Cary, NC).

The data can only be used for epidemiological research, based on clearly defined study protocols, with results published on an aggregated level, in a way that patients and care givers cannot be identified. Administrative use of data is neither allowed nor possible. Careful use of terminology is required, for example, data do not reflect the situation of people who use drugs (PWUD) in Belgium, but only of people who have entered treatment for substance use disorders.

The result of the linkage is one TDI-table with data for people who were in specialized treatment between 2011 and 2014 and tables for every of the three IMA-databases covering the period 2008 till 2017 with socio-demographic data, data about the use of reimbursed health services and data about reimbursed medication sold in public pharmacies, as well as a similar set of 10 tables for the three IMA-databases with corresponding information about the comparators. Moreover, there are two sets of 4 separate tables (one for every year between 2011 and 2014) with reference numbers linking cases with comparators: one set ordered on the NIN of cases, the other set ordered on the NIN of comparators. Table 1 gives an overview of the number of records for each table. IMA’s socio-demographic data were not yet available for 2016–2017 at the time of publication, as well as the health service data and the pharmaceutical data for 2017. For 2015 and 2016 socio-demographic data, the health service data and the pharmaceutical data were not complete because insurance agencies can provide updates and corrections up to 2 years after the end of the registration period.

**Table 1:** Number of records in TDI and corresponding number of records for every IMA table, TDI-IMA database, Belgium, 2008-2017

Year	TDI records	TDI-IMA linkage records		IMA records					
				Socio-demographic records		Health services records		Pharmaceutical records	
	cases	cases	comparators	cases	comparators	cases	comparators	cases	comparators
2008				29,956	114,546	2,832,502	4,841,577	441,524	673,262
2009				30,097	115,955	3,176,192	5,230,939	600,216	781,410
2010				30,269	117,536	3,497,624	5,503,252	645,338	794,171
2011	6011	6011	24,044	30,501	119,317	3,973,171	5,783,429	673,054	818,394
2012	7352	7352	29,408	30,574	120,401	4,704,952	6,112,367	697,780	849,270
2013	8354	8354	33,416	30,520	120,754	5,193,173	6,448,273	713,542	870,148
2014	9188	9188	36,752	30,272	120,454	5,554,960	6,754,846	725,400	888,430
2015				29,765	119,300	4,904,975	7,098,578	733,370	917,926
2016				N/A	N/A	2,794,918	4,618,602	699,002	887,280
2017				N/A	N/A	N/A	N/A	N/A	N/A
total	30,905	30,905	123,620						

N/A: not available

### Socio-demographic data

Data in Table 1 give an indication to what degree people in alcohol and drug treatment are using health services in general. Indeed, not every case in TDI was recorded every year in the socio-demographic tables, for example out of the 30,905 people in treatment between 2011 and 2014 only 29,956 were registered in the socio-demographic table in 2008, meaning that some people were not registered in 2008 in one of the health insurance agencies. Moreover, some patients were recorded in the socio-demographic tables, but without records in the corresponding health services or pharmaceutical tables, meaning that they were registered in the general health system but that they did not make use of it. As shown in Table 2, from which data for 2016 were omitted because of lack of completeness, 94.6% of the records in the TDI-database have corresponding records for every year in the IMA socio-demographic tables. For 1.6%, only 1 year is missing. On the other hand, 35 records (0.1%) have only 1 year for which corresponding data in the IMA socio-demographic tables is available.

**Table 2:** Number of years in which cases and comparators have records in socio-demographic, health service and pharmaceutical tables between 2008 and 2015, TDI-IMA database, Belgium

Number of years with linkage	IMA socio-demographic tables				IMA health service tables				IMA pharmaceutical tables			
	cases		comparators		cases		comparators		cases		comparators	
	N	%	N	%	N	%	N	%	N	%	N	%
0	0	0%	0	0%	34	0.1%	1626	1.3%	289	0.9%	4026	3.3%
1	35	0.1%	426	0.4%	73	0.2%	1578	1.3%	652	2.1%	5433	4.5%
2	75	0.2%	879	0.7%	191	0.6%	2083	1.7%	1090	3.5%	7481	6.1%
3	128	0.4%	1355	1.1%	362	1.2%	2838	2.3%	1556	5.0%	9455	7.7%
4	239	0.8%	1735	1.4%	676	2.2%	3935	3.2%	2067	6.7%	11,178	9.2%
5	282	0.9%	1746	1.4%	1226	4.0%	5780	4.7%	2908	9.4%	12,767	10.5%
6	412	1.3%	1922	1.6%	2125	6.9%	8936	7.3%	3552	11.5%	14,594	12.0%
7	501	1.6%	886	0.7%	3995	12.9%	16,870	13.8%	4739	15.3%	17,203	14.1%
8	29,233	94.6%	113,193	92.7%	22,223	71.9%	78,496	64.3%	14,052	45.5%	40,005	32.8%
total	30,905	100%	122,142	100%	30,905	100%	122,142	100%	30,905	100%	122,142	100%

### Pharmacological and health service use data

71.9% of the records in the TDI-database have corresponding records for every year in the IMA health services tables and almost half of them (45.5%) have corresponding records for every year in the IMA pharmaceutical tables. This means that more than half of the patients in the TDI-database did not use prescribed medication sold in public pharmacies at least 1 year during the reference period. On the other hand, cases purchased more medication and they addressed health services more often than comparators.

### Comparators’ data

By applying the aforementioned matching criteria, to each person in TDI four comparators were matched. However, the number of comparators is not exactly four times the number of cases. Indeed, as shown in Table 2 there are 30,905 people in treatment for substance use disorders, but only 122,142 unique comparators, meaning that as a result of the stringent matching criteria 1478 comparators have been linked to more than one case.

## Discussion

There is no extensive knowledge about healthcare pathways of PWUD before they enter specialized alcohol and drug treatment or after they have left specialized treatment. Similarly, the use and misuse of legal medication among PWUD has not been well described in international literature. The linkage of the TDI register with the IMA health insurance database allows for studying health seeking behavior and medication use of a selected but substantial group of people with substance use disorders in treatment in Belgium. To the best of our knowledge such longitudinal alcohol and illicit drug research based on pharmacoepidemiological and health service data has never been done before, although evidence shows an increasing need for such analysis [9, 20]. Illicit drug use has been identified as an important risk factor for diseases [21]. In 2015, in Belgium illicit drugs accounted for 0.6% of deaths and 1.2% of the disability-adjusted life-years (DALYs). In the same year, alcohol contributed to 3.8% of deaths and to 4.8% of the DALYs, a slight decrease compared to data for 2010 [22]. Mental health problems and infectious, pulmonary, cardiovascular and neurological diseases are more common among PWUD than among non-drug using peers [23]. These conditions may result in a higher consumption of prescribed medication among PWUD compared to non-drug using peers. At the same time, because of socio-economic and financial reasons, it is also possible that consumption of prescribed medication and/or health care use is lower among PWUD. Postponed health care often results in more costly interventions afterwards, as well as an increased risk of infections and higher morbidity and mortality [24, 25].

On the other side, the organization of the current Belgian health system, which does not include fixed general practitioners or a centralized pharmaceutical distribution register, might also encourage undesirable situations such as medical shopping whereby patients frequently go from doctor to doctor. Analyzing health care pathways can enhance the description of the existence, nature and scale of this phenomenon, to measure the use of specific medication and non-substance related medical services, before, during and after the first episode in drug treatment and to estimate under- or overconsumption of medication, compared to people who were not in specialized alcohol and drug treatment between 2008 and 2014.

Though the linked database will provide better insight in the behavior of people with substance use disorders, there are several limitations. On the one hand, the TDI-database has some specific restrictions as reported by Antoine et al. [11]. Firstly, it is based on self-reported data, which implies the risk of misreporting. Secondly, registration of the NIN is not mandatory and it is missing for approximately 33% of data in TDI. As a result, one third of the patients in the TDI-database are not linked to the IMA-database (further analysis will be done to study the differences between both groups to see if this results in a selection bias). Thirdly, TDI is a tool for incidence of drug treatment episodes. For the linkage the first episode of patients within the reference period was selected, which means that patients might have been in treatment before. This is only indirectly known through a self-reported variable in the TDI-database (ever been in treatment for substance abuse before: yes/no (Q19)) [11].

On the other hand, the IMA socio-demographic, medication and health services databases were not initially created for epidemiological purposes but remain administrative tools. They reflect only medical care that is reimbursed by the social security. This has two important consequences: firstly, as this reimbursement procedure for use of health services is not an automatic process yet, it remains the responsibility of the patient to request reimbursement, hence resulting in a possible loss of data. Secondly, not all health care is reimbursed and therefore part of the health seeking behavior related to care or the consumption of specific medication remains unknown. Indeed, health care services such as those provided by psychologists or physiotherapists are not reimbursed. Since people with substance use disorders make frequently use of these services, this information is clearly missing. Data also do not cover over-the-counter (OTC) drugs, prescribed but not reimbursed medication such as benzodiazepines, or self-medication. Besides, data on prescribed medication is based on DDD, which is a theoretical construct, rather than a directly observed indicator such as Prescribed Daily Doses (PDD) or Consumed Daily Doses (CDD), which are reflecting better actual consumption [6]. It is classified by a CNK-code and ATC-code, but as a lot of medication is used for a wide variety of diseases and illnesses, it is usually not possible to know the exact clinical cause for the prescription of certain medication. In the Norwegian Prescription Database for instance, ICD-10 codes can be registered, which in some cases may function as a proxy of diagnosis [1].

Off-label use of medication - the prescription of medication in a manner different from that approved by the regulating authorities - will not be revealed through the TDI-IMA-database, nor does the database include data on drugs provided in prison. Prescriptions do not give any information about non-compliance, which is a common phenomenon [24, 26, 27]. The frequency of use is unknown (excessive indulgence on medication, known as ‘binging’, will remain unnoticed), and also the procurement of prescriptions with the intention to resell medication will remain undetected. Finally, the results will only show medication that is sold through registered pharmacies. As previously reported [28, 29], many PWUD purchase medication through online pharmacies or from drug dealers.

Finally, since comparators are matched on age, sex and place of residence, comparison of results will only be possible between people in treatment for substance use disorders and their peers who are not in specialized treatment. Generalization to the general population will remain impossible.

## Conclusions

In several European countries, pharmacoepidemiological databases [1, 2, 4–6, 20] and the Treatment Demand Indicator [11] are both available. Based on the NIN it was possible in Belgium to link a national pharmacoepidemiological and health services database to data about PWUD. This combined database will allow a better understanding of (1) the care pathways followed by people who end up in alcohol or drug treatment, (2) the medication they have used and (3) how the health seeking behavior evolves afterwards. The third European TDI protocol, which is in place since 2015, has increased the potential for research on substance use disorders through linkages of TDI with external databases. Based on this protocol, the presented linkage could be used in other European countries as well.

## Abbreviations

EMCDDA: European Monitoring Center for Drug and Drug Addiction
EU: European Union
IMA: Health Insurance Agency (InterMutualistisch Agentschap – Agence InterMutualiste)
NIHDI: National Institute for Health and Disability Insurance
NIN: National Identification Number
TDI: Treatment Demand Indicator
